# Experimentally Simulating Paternity Uncertainty: Immediate and Long-Term Responses of Male and Female Reed Warblers *Acrocephalus scirpaceus*

**DOI:** 10.1371/journal.pone.0062541

**Published:** 2013-04-29

**Authors:** Herbert Hoi, Ján Krištofík, Alžbeta Darolová

**Affiliations:** 1 Konrad Lorenz Institute of Ethology, Department of Integrative Biology and Evolution, University of Veterinary Medicine, Vienna, Vienna, Austria; 2 Institute of Zoology, Slovak Academy of Sciences, Bratislava, Slovakia; Hungarian Academy of Sciences, Hungary

## Abstract

In many socially monogamous species, both sexes seek copulation outside the pair bond in order to increase their reproductive success. In response, males adopt counter-strategies to combat the risk of losing paternity. However, no study so far has tried to experimentally prove the function of behaviour for paternity assurance. Introducing a potential extra-pair partner during the female fertile period provides a standardised method to examine how pair members respond immediately (e.g. increase mate guarding or copulation frequency) or long term (e.g. later parental investment and paternity uncertainty). In this study on a socially monogamous passerine species, we experimentally confronted pairs of reed warblers with a conspecific male (caged male simulating an intruder) during egg-laying. Our results revealed that occurrence of an intruder during that period triggered aggression against the intruder, depending on the presence of the female. The male territory owner also attacked the female partner to drive her away from the intruder. Thus territory defence in reed warblers also serves to protect paternity. The increase in paternity uncertainty did not affect later paternal investment. Paternal investment was also independent of the actual paternity losses. In females, the experiment elicited both, immediate and long-term responses. E.g. female copulation solicitations during the intruder experiment were only observed for females which later turned out to have extra-pair chicks in their nest. In relation to long term response females faced with an intruder invested later less in offspring feeding, and had less extra-pair chicks in their nests. Extra-pair paternity also seems to be affected by female quality (body size). In conclusion female reed warblers seem to seek extra-pair fertilizations but we could demonstrate that males adopt paternity assurance tactics which seems to efficiently help them to reduce paternity uncertainty.

## Introduction

In many socially monogamous species, both sexes frequently seek copulations outside the pair bond to increase their reproductive success [1]. As a consequence, males in particular adopt pre- and post-copulatory strategies to respond to the risk of cuckoldry. The most common pre-copulatory strategy is to guard the fertile female and in this way also prevent her from copulating with rival males [1]–[7]. Another pre-copulatory strategy is territory defence [8]–[11]. By excluding other males from the territory males may at the same time exclude them from copulating with their females. Frequent within-pair copulation is recognised as the most common post-copulatory strategy to prevent extra-pair fertilisations [12]–[15]. Post-copulatory responses include direct physical punishment of the female [16] or indirect retaliation by reducing paternal care [17], [18]. There is, however, not much evidence for a reduction in paternal care, which may have several causes. Adjusting paternal investment according to the risk of cuckoldry would mean that a male is able (i) to accurately determine the risk of cuckoldry and (ii) to discriminate between own and extra-pair nestlings. Whether males are able to evaluate paternity certainty properly is, however, not yet confirmed and a wrong classification of a male’s genetic offspring would certainly reduce its fitness. In contrast, females may only seek extra-pair copulations when they expect their males not to retaliate for their extra-pair behaviour. In fact, one would even expect a positive relation between extra-pair paternity and male investment in offspring [18], which was found at least in one interspecific comparison [19]. In this study we experimentally simulated an increased risk of paternity uncertainty in a passerine bird species namely the reed warbler (*Acrocephalus scirpaceus*) by confronting a pair with an extra male for two 20-minute sessions during the fertile period of the female and to examine their immediate response (e.g. reaction against the intruder and the female mate, or female extra-pair behaviour) and long-term consequences (e.g. adjustment of offspring feeding investment). Intrusions are normally very short incidences, because as soon as a territory owner detects an intruder it usually takes a few seconds until the intruder is displaced. The pair members should therefore perceive our confrontation experiment as a significant disturbance. Male and female perception of such an intruder confrontation might be different, however. A female may perceive her partner as weak if he is not able to displace the intruder immediately. Consequently one would expect extra-pair paternity to be higher in the experimental than in the control group if the female is under control of the fertilization process. A male perceives an intrusion as an increased risk of paternity uncertainty, in particular due to the long persistence of the conspecific male. Consequently one would expect extra-pair paternity to be lower in the experimental group because males may as a response invest more in mate guarding and territory defence. The reed warbler is a good model system for this investigation. It is a socially monogamous, territorial species with biparental care. Breeding starts in April [20] and extra-pair paternity is known [21]. Male contribution to offspring care is higher than in related warbler species [20], indicating the importance of paternal care. Mate guarding seems to be the principal paternity assurance tactic, because during the fertile period males spend most of their time close to their mate [21]. From arrival until the end of the fertile period, which stops after females lay their penultimate egg, males are territorial and try to repel any intruder [20], [21]. With the start of incubation territory defence usually collapses [21], [22]. Hence, territorial behaviour could be an additional paternity guard and aggressiveness against conspecific males may help to prevent them approaching the female and consequently lower the risk of extra-pair fertilisations. Thus, intensive investment in paternity guards may be one reason for their relatively low rate of extra-pair paternity (e.g. 6% of all young in 15% of all broods, see [21]).

Here we want to examine (i) how males and females react when experimentally confronted with an intruder during the female fertile period, (ii) whether this has any consequences on their later parental investment, and (iii) whether it affects paternity uncertainty. For this reason each experimental pair was confronted with a conspecific male on two consecutive days for 20 min when egg-laying started (one or two eggs in the nest).

## Methods

The study was done at Veľké Blahovo fishponds (Slovakia, 48°03′09″ N, 17°35′38″ E). Reed (*Phragmites australis*) and cat tails (*Typha latifolia* and *T. angustifolia*) are the dominant plant species. Nests are usually built over flooded ground, up to a depth of 1 m. We first mapped male territories, than determined female arrival and finally used nest building behaviour to determine nest site location.

### Design of Confrontation Experiment

The experiment started during the fertile period of the female, i.e. with the first or second egg. Each confrontation experiment consisted of two 20-min exposures of a conspecific male, each on two successive days. The ‘intruder’ male was offered in a cage (70×50×50 cm). He was hand-reared and acclimatised to the cage so that he behaved naturally. The intruder cage was placed about 3 m from the nest and a loudspeaker was placed on top of the cage. Observers were hidden in a blind about 10 to 20 m from the cage. During the 20 min presentation, the song of a randomly selected male (out of a pool of song records of ten males) from this area, recorded the year before, was played back.

In the control experiments we did exactly the same and the only difference was that the cage was empty and no observations were made from the blind.

During the 20-min observation we recorded the following parameters for males: (i) presence of the territory owner during the confrontation experiment, (ii) whether the territorial male arrived near/at the intruder cage (0 to 1 m of the cage), (iii) male attacks on the caged male, (iv) aggressions against the female partner, (v) occurrence of male song, (vi) duration of male song and finally (vii) male nest visits during the confrontation experiment.

In females we recorded: (i) female presence during the experiment and (ii) female copulation solicitation behaviour.

In total we performed confrontation experiments with 31 pairs, for 24 of which we had complete behavioural observations. Experimental and control nests were randomly selected.

### Morphology and Blood Sampling

Male and female territory owners were trapped with mist nets during the feeding phase. At that point a small blood sample was taken from the brachial vein (ca. 30 µl) and the following morphological variables to the nearest 0.1 mm recorded: tail-, bill-, tarsus length using callipers and body mass using a digital scale (to the nearest 0.1 g). Birds were also individually colour-ringed for identification during the feeding observations.

For paternity analyses a blood sample (ca. 30 µl) was also taken from 6-9-day-old nestlings and the same morphological variables (except tail length) were determined for each chick in the nest. For paternity analyses we have been able to collect blood from 29 families (fifteen families belonging to the experimental and fourteen families to the control treatment). For these families feeding observations were based on one-hour observation bouts (two or three observation sessions for each nest). Feeding observations were done in the early morning between 07.00 and 11.00 on two or three consecutive days (7-9-day-old nestlings). Parent feeding rate did not vary in relation to nestling age (Repeated measures ANOVA, F = 0.41, p = 0.524, df = 1,28, each nest entered with two feeding protocols –feedings/h - for different days).

### Statistical Analyses

We tested whether data meet assumptions for normality and applied statistical tests accordingly.

Using an Oneway ANOVA we found no difference between experimental and control nests in any male or female morphological variable investigated including bill-, wing–, tail-, and tarsus length and body mass (p>0.3 for all variables of both sexes). There was also no difference in start of laying (p>0.7) and clutch size (p>0.2) between experimental and control nests. Thus there are no obvious quality differences between the individuals used in the two treatment groups. To evaluate the effect of the experiment on nestling condition we compared the average nestling body mass/nest between control and experimental nests using wing length as covariate.

Differences in extra-pair paternity between broods with experimental and control treatment were analysed using generalized linear models using a quasi-likelihood function to correct standard errors of parameter estimates and consequently p-values to avoid overdispersion.

When examining immediate response, to avoid pseudo replication and habituation we used only the first protocol for the analyses.

A 2×3 exact test after Freeman-Halton [23] was used to determine the relationship between female presence and male aggression intensity towards the intruder. Male aggression intensity was expressed in three ordinal categories were intensity of aggression increases with each category (aggression intensity in category 1< category 2 and category 3): (category 1) no reaction - males did not appear near the intruder (male not seen within 3 m of the intruder cage), (category 2) medium reaction - males approached the cage, and (category 3) strong reaction - males attacked the intruder.

To examine whether male or female quality is related to offspring feeding rates a stepwise procedure was used for model selection in a multiple regression analyses. Morphological measurements and body weight were used as independent parameters and feeding rates as the dependent variable. Regression analyses were done separately for each sex. To examine whether extra-pair paternity is related to individual quality we used a stepwise discriminant function analyses with morphological measurements as independent parameters and the presence/absence of extra-pair nestlings as grouping variable.

For statistical analyses we used SPSS statistics version 20.

### DNA Extraction and Amplification

DNA was extracted from the blood samples using DNeasy Tissue Kit (Qiagen). We used four to seven microsatellite primers to assign paternity – Ppi2 [24], Ase 18, Ase 34, Ase 48, Ase 58 [25], Fhu2 [26], Pca3 [27]. The set of microsatellite primers (originally developed for other species) was previously tested and optimized for the reed warbler [28], [29]. Amplification by polymerase chain reaction was performed according to the following condition: initial denaturation 95°C –15 min, followed by 35 cycles 94°C –30 sec, 58°C –90 sec, 72°C –90 sec, and finally 72°C –10 min. Each 12.5 µl reaction consisted of 1–20 ng DNA (2 µl), 6.15 µl H_2_O, 2.5 µl reaction buffer containing 7.5 mM MgCl_2_ (Finnzymes), 1.25 µl dNTPs, 0.25 reverse primer, 0.25 forward fluorescent primer, and 0.1 µl Phusion DNA polymerase (Finnzymes). Fragment analysis was performed by use of Sequencer CEQ 8000 Beckman.

We defined any chick with two or more allelic mismatches with the social father as an extra-pair offspring. All offspring were profiled at a minimum of four loci. The number of analysed individuals, size range, number of alleles and observed heterozygosity for each primer used are summarised in Table 1.

**Table 1:** Number of analysed individuals, size range, number of alleles, observed heterozygosity, and null allele frequency estimate at each microsatellite locus.

Locus	Number of individuals	Range (bp)	Number of alleles	Observed heterozygosity	Null allele frequency
Ase18	163	158–178	10	0.890	0.0357
Ase34	170	230–262	16	0.894	0.0186
Ase48	163	260–380	19	0.816	0.0631
Ase58	167	186–258	11	0.838	0.0472
Fhu2	162	131–148	8	0.809	0.0313
Pca3	144	133–158	11	0.859	0.0388
Ppi2	166	242–280	23	0.868	0.0563

### Ethics Statement

All procedures regarding observational and experimental field study were conducted according to the respective legislation of the Slovak Republic and following the conditions and guidelines approved by the Ministry of Environment of the Slovak Republic (permit number 1443/04).

The study on the protected Reed Warbler was conducted in a special protected area (SPA) within the network of NATURA 2000. Permission to enter and to work with this species was issued by the Ministry of Environment of the Slovak Republic (permit number 1443/04). The experimental time was set to a minimum for the purpose of the study. Routine procedures (e.g. trapping, ringing, blood taking – for details see methods) were done by very experienced persons and therefore could be reduced to a minimum time. All experimental nests have been successful (all nestlings fledged) and there were no differences in body weight between treated and control nestlings (see results).

## Results

### Male and Female Immediate Response to an Intruder

All males approached the intruder up to a distance of about 2 m and most of the males (79.2%; 19/24) jumped directly onto the cage with the intruder.

**Figure 1:**
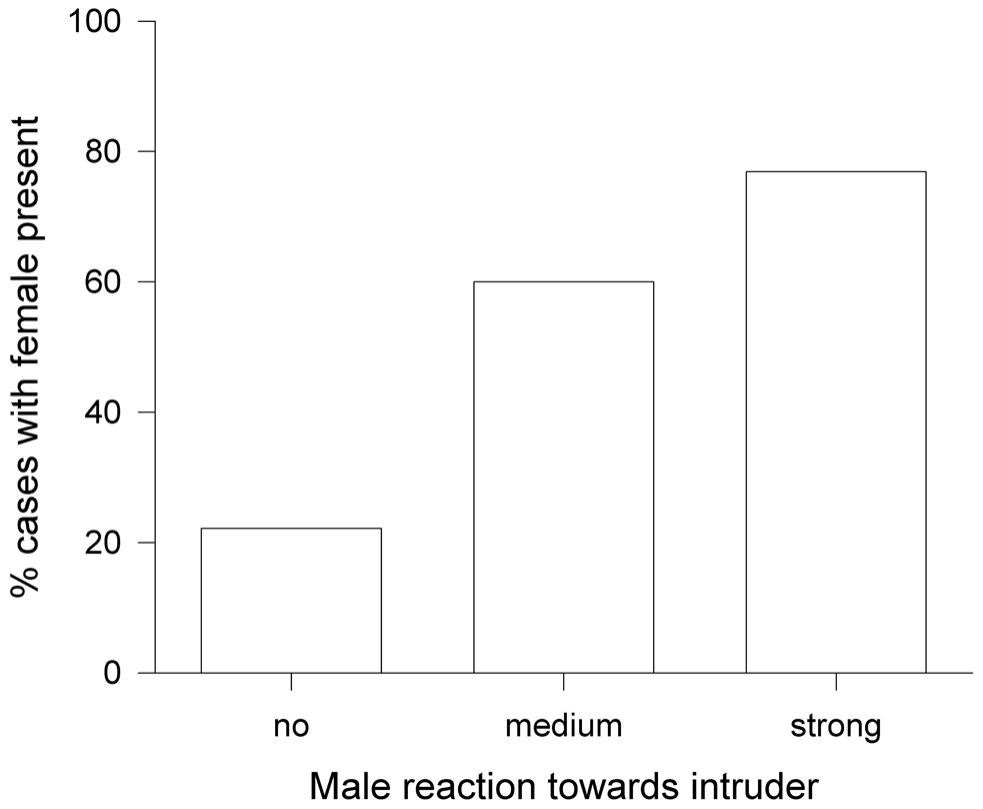
Male aggressions intensity towards intruders in relation to female presence. Relationship between strength of male reaction towards the intruder (no reaction = no, approach = medium and attack = strong reaction) in relation to whether the female was present (%).

The strength of a male’s response towards the intruder was influenced by the presence of the female (see Figure 1). Strong aggression (e.g. attacking the intruder) was significantly more frequent when the female was present (Freeman-Halton exact test, p = 0.038).

Whether a male attacks an intruder was also significantly related to female behaviour (two sample proportions - test, z = 2.01, p = 0.044, n = 11, 13). 64% of the males attacked the intruder when females solicited copulations near the intruder in comparison with only 23% of the males, in the absence of female copulation solicitations. During the experiment males also attacked their female partner, but these attacks have not been significantly related to females’ copulation solicitations (two sample proportions - test, p>0.3, n = 24).

We found no morphological difference between males which attacked the intruder or males which did not (p>0.3 for all morphological parameters investigated; see Methods).

In contrast to males, only 58.3% (14/24) of the females were observed during the experiment. Thus almost a half of the females did not even appear near the intruder.

Furthermore no female aggression was observed against the intruder or the social partner. Some females even solicited copulations near the intruder (45.8%; 11/24). In all cases, however, the male partner was also present, and therefore it was unclear to whom copulation solicitations were actually directed.

### Consequences for Male and Female Investment in oOffspring Feeding

We found no difference in the total number of feedings/h (male and female feedings pooled) between experimental and control nests (ANCOVA; F = 0.37, p>0.5; df = 1,28 covariate (number of nestlings): F = 0.77, p>0.3, df = 1,28). Also, the average nestling body mass/nest did not differ between experimental and control nests (F = 0.23, p>0.6; df = 1, 28; covariate (wing length): F = 3.09, p = 0.09, df = 1, 28).

**Figure 2:**
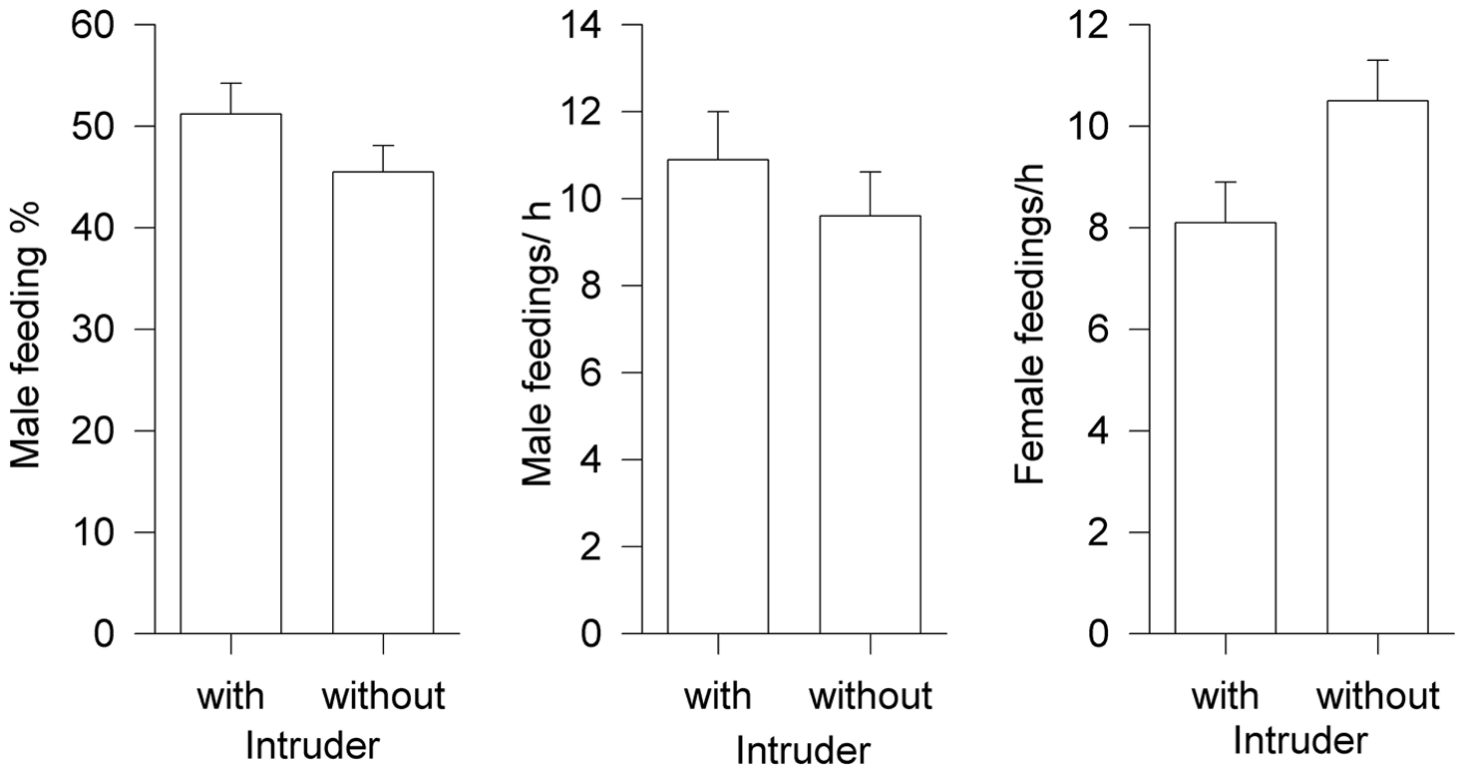
Feeding investment of pairs with (n = 15) or without an intruder (n = 14) during the fertile period. Given is male contribution in % (left diagram), male feedings/h (middle diagram) and, female feedings/h (right diagram) in relation to the presence of an intruder.

Examining the proportion of male/female feedings revealed a significant difference between control and experimental nests (Figure 2). Males from experimental nests seem to contribute significantly more (F = 4.61, p = 0.043, df = 1, 28; covariate (number of nestlings): F = 0.001, p>0.9, df = 1, 28). Examining the absolute feeding rates of males and females separately revealed, however, that feeding rates of experimental males did not differ in comparison with control males (F = 0.08, p>0.7; df = 1, 28; covariate (number of nestlings): F = 0.01, p>0.9, df = 1, 28) but female feeding rates did vary, in that control females fed their offspring significantly more frequently than females faced with an intruder (F = 4.01, p = 0.049, df = 1, 28; covariate (number of nestlings): F = 0.04, p>0.5, df = 1, 28) (Figure 2).

Male feeding effort correlated with male morphological features. Male wing and tail length were subjected to stepwise multiple regression analysis with average male feeding rates/h as the dependent variable (regression model: F = 6.33, p = 0.006, r^2^ = 0.34, df = 2, 26). The partial correlation coefficient suggested that males with longer wings (r_part_ = 0.45, p = 0.018) and longer tail feathers (r_part = _0.41, p = 0.03) invested more in offspring feeding.

Females’ morphology in contrast did not explain their feeding investment since no variable entered the model (overall regression model: F = 0.4, p>0.5, r^2^ = 0.01, df = 26). Furthermore, female investment in offspring feeding did not differ depending on the behaviour of the female during the confrontation experiment, e.g. female present or absent (p>0.3, n = 24) or female solicitation of copulation or not (p>0.7, n = 24).

### Does Intruder Presence Affect Extra-pair Fertilizations?

**Figure 3:**
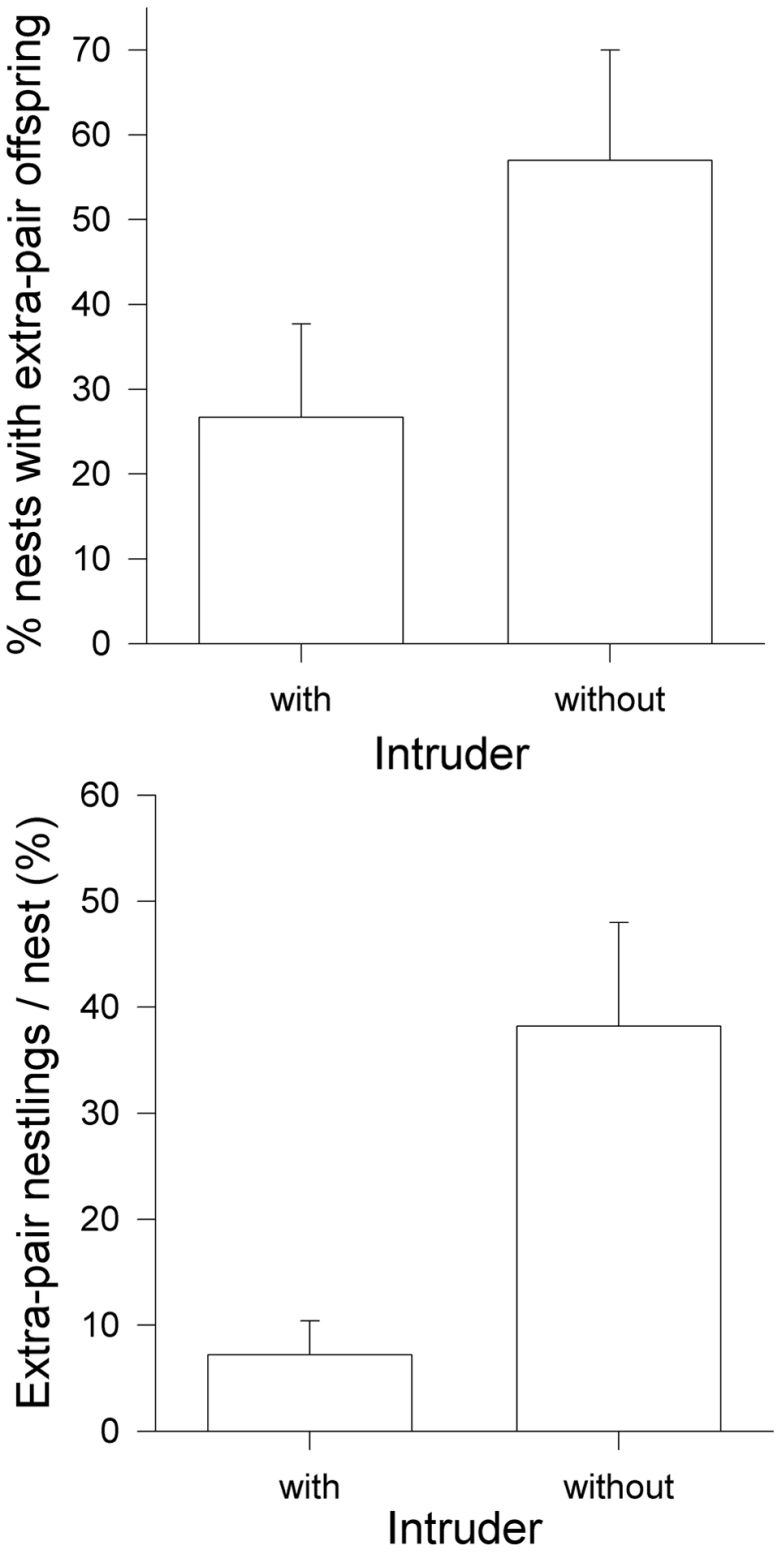
Extra-pair paternity in relation to the presence of an intruder. Proportion of nests with or without extra-pair young (upper graph) and average proportion of extra-pair nestlings (lower graph) in relation to the occurrence of an intruder during the fertile period.

Extra-pair fertilisations occurred in 44.45% of the nests (n = 29 nests) and 21.1% (n = 109 nestlings) of all nestlings were extra-pair offspring. We found a significant difference in the frequency of extra-pair paternity in relation to the experiment. More nests contained extra-pair chicks in the control nests. This difference is not significant (two sample proportions - test, z = 1.67, p = 0.09, n = 15,14), but the proportion of extra-pair nestlings/nest were significantly higher in experimental than control nests (generalized linear model with quasi-binomial error structure: t_27_ = −2.49, p<0.01, n = 15,14) (Figure 3).

Male behavioural responses to an intruder (for description of behavioural parameters, see Methods) did not show any significant relationship with the occurrence of extra-pair nestlings in the nest or the proportion of extra-pair nestlings in each nest (p>0.3 for all). Moreover, male feeding investment (number of feeds/h) did not vary in relation to the occurrence and frequency of extra-pair paternity (p>0.4 for both). Finally, no morphological variable investigated (see Methods) entered a stepwise discriminant function analysis comparing males of nests with or without extra-pair nestlings.

There was a non-significant tendency whereby female solicitation behaviour was related to extra-pair paternity. Almost half (42.8%) of the females which performed copulation solicitations tended to have extra-pair nestlings in their nest whereas no female who did not show this behaviour had extra-pair nestlings (F = 3.12, p = 0.1, df = 1, 23).

Out of all female morphology variables investigated, only tarsus length entered a stepwise discriminant function analysis. Thus females with extra-pair chicks in their nest had significantly longer tarsi than faithful females (females with: mean = 24.36 mm ±0.26, female without: mean = 23.88±0.24; F = 6.3, p = 0.019).

## Discussion

There are two approaches to investigating the importance of the presence of the male partner in relation to female extra-pair behaviour. Most studies try to detain the partner during the fertile period and indeed have found support for the importance of mate guarding as a paternity guard [30]–[32]. This method has, however, several shortcomings. E.g. from the female point of view, an increase in extra-pair paternity could be differently explained. If the male disappears, the female may interpret this on one hand as her male partner has deserted or was killed which may consequently induce females to seek for new mates. On the other hand, females may start to search for their missing mate which could increase the risk of harassment by neighbouring males. Females, when missing their mate may also utter distinct contact calls which at the same time signal to other males that the female is unguarded (own unpublished observations). Moreover, it is difficult to interpret whether females use male absence actively to seek extra-pair copulations or whether females suffer male harassment and coercion and therefore suffer forced extra-pair copulations [33]–[37]. On the other hand, there are at least a few studies which induced an apparent risk of paternity loss by offering a decoy [38]–[41]. Difficult to comprehend in such an approach is the perceived risk represented by, for example, offering a caged conspecific. Beneficial of such an approach may be, that it provides additional information, e.g. on male and female direct response to approaches of potential extra-pair partners. Territory intrusions by conspecific males are usually short and hidden events. They occur irregularly, and are hence difficult to predict and observe. Similarly, extra-pair copulations are rarely observed even in species with very high rates of extra-pair paternity [42]. Consequently observational data are scarce for such intrusion events during the fertile period and for male and female behaviour. Relating the response of pair members to the time when the experiment is performed (e.g. mating, fertile, incubation period), or strength of the intruder experiment (e.g. length of the challenge, distance to the nest, female, etc.), may provide further insights. In line with this the results of our experiment confirm the importance of males’ strategies to protect paternity. A role of mate guarding in this species was already suggested in earlier studies [21], [43], [44]. Our results show that during the fertile period males usually try to chase an intruder out of the territory but, more importantly, we could demonstrate that intensity of aggression against the intruder depends on whether the female partner was present (Figure 1). A similar result was found for meadow pipits [45]. In their study, however, the experiments were spread over the whole mating period and hence male behaviour could also be interpreted as female defence against intruders, to reduce the risk of mate switching and female desertion with an intruder [46]. In the closely related, socially monogamous moustached warbler *Acrocephalus melanopogon*, mate guarding does not seem to be an efficient male paternity guard. This is evidenced by the rather high rate of extra-pair paternity and the fact that sometimes an extra male appears during the first brood and the female switches to this male for a second breeding attempt [47]. In our experiments females were fertile (in egg-laying phase), and we know from aviary observations that females continue copulating until the last egg is laid (unpublished observations). As soon as females started egg-laying, the risk of female desertion was low. The interpretation of our results as anti-cuckoldry behaviour is further confirmed by the fact that males, when unable to repel an intruder, even attack their own female partner, in particular when they start to solicit copulations near the intruder (see results). Thus territorial behaviour of male reed warblers probably operates as an additional paternity guard. That males switch from mate guarding to more female-focused defence during the fertile period was also demonstrated for stitchbirds *Notiomystis cincta* [8]. A function as a paternity guard in our male reed warblers is further confirmed by the facts that the intensity of territory defence (i) almost ceases after the fertile period of the female (e.g. when females start incubating), and (ii) increases from the nest building to the egg-laying period [22], [48]. The change in the intensity of territory defence is also mediated by song production, which is shown to have a territorial function as well [49], [50] and almost completely disappears until clutch completion. The variation in male response, depending on the risk in a situation, suggests that males are able to fine-tune their investment into paternity assurance behaviour [8], [32], [41], [51]–[53].

In contrast to the immediate response, our study revealed no male long-term effect. E.g. male feeding investment is not influenced by actual and apparent paternity losses, as also found by [30] for western blue birds *Sialia mexicana* or [54] for dunnocks *Prunella modularis* but see [18]. The occurrence of an intruder for a relatively long time during the fertile period and assuming this is perceived as an increased risk of paternity, had no obvious effect on male investment in offspring feeding. Actually males tended to invest slightly more in their offspring when they suffered an intruder during the fertile period of their mate (Figure 2). Furthermore, males did not change feeding investment in relation to whether they suffered from extra-pair chicks. As in earlier studies [54], however, it remains unclear how to explain this negative result. The only influential factor in relation to male feeding investment was male size. We found a positive relationship between male feeding rates and wing- and tail-length which might be a quality (age) indicator [54], [55]. On the other hand we found no evidence that males would be able to discriminate between own and extra-pair nestlings or at least their chick feeding rules are not influenced by the possibility of suffering from extra-pair paternity. On the other hand assuming that mate guarding is costly [2] it is likely that bigger males invest more in mate guarding [2], [5].

Female immediate response on the other hand seemed to be much weaker and variable as only about 60% of the females even appeared near the intruder and they did not show any aggressive behaviour. In contrast several females even showed sexual behaviour. This variation in females is not a direct result of our experiment but in fact our results suggest that it is bigger females which seem to be more promiscuous. There are several factors, like age, experience [56] or personality [57] which may be responsible for variation in behaviour and eventually also in extra-pair paternity.

Also in relation to the long term response females behaved differently. Faced with an experimental intruder they showed later significantly lower feeding rates than control females. One possible explanation for this result is that a female perceives her own partner as weak when he is not able to displace the intruder in a proper way. Theory predicts that females should allocate their resources according to male attractiveness or quality [58]. Consequently females may reduce their feeding investment when males are perceived to be unable to repel an intruder, as we observed for our experimental group. This effect should be in principle limited to the females actually present during the experiment and consequently the results in relation to the response may become stronger if our analyses are restricted to those females. On the other hand it is unlikely that females which did not come into view are not fully aware of the situation, particularly because reed warbler territories are very small.

In contrast to extra-pair behaviour, female quality in terms of morphology and body mass did not seem to influence maternal feeding investment and female behaviour during the intruder experiment was also no indicator of later investment.

However, here we could show that the occurrence of an intruder during the fertile period affects extra-pair paternity. Extra-pair paternity in our population is relatively high [21] but we found extra-pair paternity to be less frequent with the occurrence of an intruder. The most likely explanation for this result is that males, as a consequence of potential stress owing to the persistence of the intruder, increased their mate guarding effort. If the significant reduction in paternity losses is owed to mate guarding one would expect a trade-off between investment in mate guarding assuming mate guarding is costly [2] and other behaviours, otherwise all males should invest more in mate guarding. In contrast, our data suggest that female behaviour, particularly solicitation behaviour during the fertile period, and female intrinsic quality, namely female body size, influence whether they seek extra-pair copulations or not.

In conclusion our results suggest that both sexes responded to the experimentally induced intruder. Females show immediate - as well as long-term responses (e.g. they actively seek extra-pair copulation). Female intrinsic quality seems to have a significant effect on how females behave during intruder confrontations and extra-pair paternity. Females also change later offspring investment depending on the presence of an intruder. Males try to lower the risk of paternity uncertainty by immediately responding to potential extra-pair males or punishing their female partner. There is no evidence, however, for long-term changes, e.g. that males punish their females by desertion or reducing parental care. Males have different strategies including mate guarding and territorial defence to prevent extra-pair copulations.
